# National Association for Promoting the Welfare of the Feeble-Minded

**Published:** 1905-06-03

**Authors:** 


### NATIONAL ASSOCIATION FOR PROMOTING THE WELFARE OF THE FEEBLE-MINDED.

With their president, Lady Frederick Brudenell Bruce in the chair, the National Association for Promoting the Welfare of the Feeble-minded held their annual meeting at Denison House, Vauxhall Bridge Road, on May 26th. Rising to propose the adoption of the report, Lady Brudenell Bruce spoke of the Association's many causes for congratulation in the successes which had been accorded to their work. Foremost was the grant of the Royal Commission which they had worked unremittedly to obtain. She was very glad to say, too, that the Association had now succeeded in freeing themselves from their liability to the bank; they did not want to become again in debt, and it was earnestly hoped that the public would come forward with generous assistance.

The Bishop of Stepney seconded the motion, and said that from experience he could testify to the great necessity for the Association's work. He knew of three classes of mentally and morally deficient persons. First, men and women who resided in the lowest type of common lodging-house and who almost invariably propagated their unhappy species and added to the world's population, by large families of children. Second, the unfortunate type of boy constantly drifting into prison. Lads who were arrested and sent to reformatories, only in course of time to be dismissed and almost immediately rearrested. It was a strange fact, continued the speaker, that boys suffering most from mental and moral deficiency often showed the keenest aptitude for learning the Bible. To a certain extent, also, they were smart under training, but they suffered from an extraordinary perversion of will-power. The third class were mentally and morally deficient girls—that wretched class who year by year were drifting hopelessly onward, almost spending their lives in propagating their own misery. The present course of treatment—of education for these boys and girls was wrong, education only made them more dangerous. Useful and self-supporting citizens they could never be; but their lives might at least be happy and harmless if proper measures for care and sequestration were taken.

Mr. Francis Galton, in supporting the motion, said the Association were greatly to be congratulated on their combination of philanthropy and research. It was of enormous importance that the life-histories of the feeble-minded should be known, and not only their personal records but those of their parents, grandparents, and relations. The work of obtaining this information would have to be both thorough and exhaustive—perhaps it appeared almost an impossible undertaking; but if their helpers would each do a little, they would soon find their efforts had excellent results. The breed of the nation should certainly be improved, and measures taken to prevent the propagation of feeble-minded persons.

The motion having been carried, Sir William Chance proposed a vote of thanks to the speakers, and referred to the power conferred by the Act of 1899 for providing for feeble-minded children.

Mr. A. A. Allen seconded the motion and said that as chairman of the sub-committee of the London County Council, which was responsible for the education of mentally deficient children, he saw how very great was the need of the Association's voluntary work. Unfortunately these children at present left school at sixteen and went out into the world, but he had every reason to hope that in the future the County Council would be able to come to some arrangement with the National Association on this most vital question of the after-care of the feeble-minded.

The Bishop of Stepney having replied to the vote, the proceedings were terminated by a vote of thanks to Lady Brudenell Bruce for presiding.

